# Nanomaterials for the Capture and Therapeutic Targeting of Circulating Tumor Cells

**DOI:** 10.1007/s12195-017-0497-4

**Published:** 2017-07-20

**Authors:** Zhenjiang Zhang, Michael R. King

**Affiliations:** Department of Biomedical Engineering, Vanderbilt University, Nashville, TN 37235 USA

**Keywords:** Cancer, Metastasis, Circulating tumor cells, Nanomedicine, Nanomaterials, Nanoparticles

## Abstract

Circulating tumor cells are a hallmark of cancer metastasis which accounts for approximately 90% of all cancer-related deaths. Their detection and characterization have significant implications in cancer biology and clinical practice. However, CTCs are rare cells and consist of heterogeneous subpopulations, requiring highly sensitive and specific techniques to identify and isolate them with high efficiency. Nanomaterials, with unique structural and functional properties, have shown strong promise to meet the challenging demands. In this review, we discuss CTC capture and therapeutic targeting, emphasizing the significance of the nanomaterials being used for this purpose. The next generation of therapy for metastatic cancer may well involve capturing and even directly neutralizing CTCs using nanomaterials.

## Introduction

**Figure 1:**
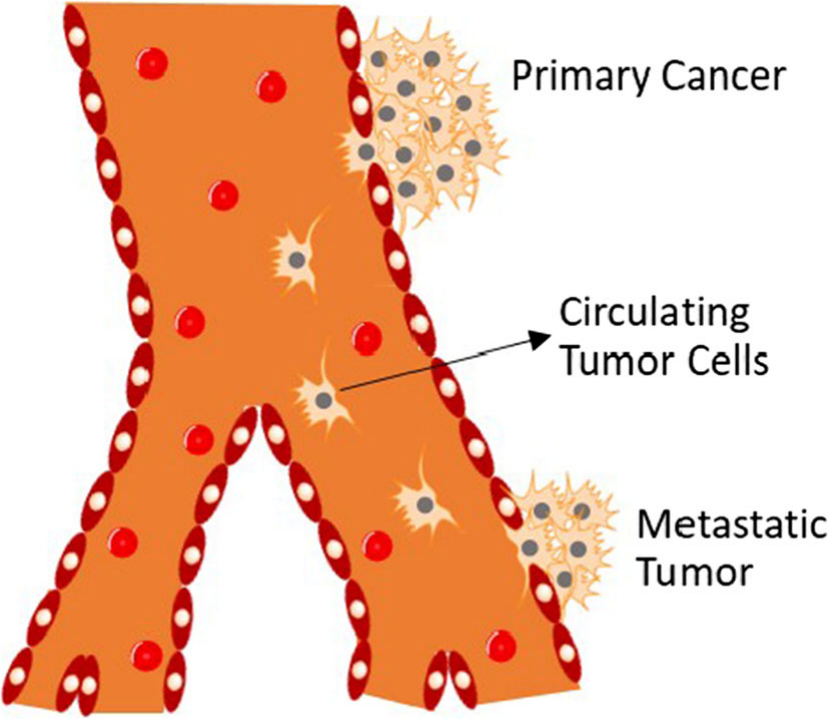
Schematic view of the distant metastatic progression showing CTC transit: CTCs break away from the primary tumor, intravasate into the blood circulation, travel, and extravasate into a distant site where they may ultimately develop metastasis or enter into dormancy.

Cancer metastasis is the major cause of cancer morbidity and mortality, accounting for approximately 90% of all cancer-related deaths.^101^ The progression of metastasis is complex and involves multiple sequential and interrelated steps and multiple biochemical events with much remaining to be elucidated.^50^ One necessary step in distant metastasis that has come to be recognized is the transport of cancer cells that break away from the primary site and travel through the bloodstream of cancer patients (Fig. 1).^22^ Tumor cells in the circulation, so called “circulating tumor cells” (CTCs), will mostly die in transit as they are vulnerable to death induced by shear stress and turbulence by immune cells.^82^ Only a very small number of CTCs will ultimately extravasate from the circulation and seed the growth of a secondary tumor in a distant organ.^82^ It is these few surviving and proliferating cells that account for cancer mortality. There is a clear direction of interest in cancer research to isolate and characterize, as well as directly kill CTCs in the circulation or prevent them from developing metastases. The prognostic significance of CTC detection has been clinically recognized in several types of cancer, including breast, prostate, colon, lung cancer and melanoma.^81^ Characterization of CTCs is also informative for the monitoring and prediction of the response to ongoing therapy.^127^ In addition, the isolation of CTCs and subsequent genomic analyses show strong promise for early cancer detection because they are sometimes released during early stages of tumor development and have been found in patients with relatively small primary tumors (e.g., breast cancer).^70^ Furthermore, molecular profiling of CTCs may provide insights into mechanisms of cancer progression, which could potentially lead to the discovery of new therapeutic targets.^114^

CTC detection, capture or direct therapeutic targeting in the circulation, however, is inherently very challenging. The major challenge is that CTCs are very rare, with numbers as low as one CTC in 1 mL of patient blood mixed with billions of red blood cells (RBCs) and millions of white blood cells (WBCs).^52^ Another key challenge is that they represent a heterogeneous population of cells with diverse biological and molecular characteristics, often different from those of the respective primary tumor.^31^ Hence, efficient isolation requires a high level of simultaneous specificity for various CTCs and the ability to handle a very small number of cells.^60^ As a result, significant advancement in this area has only been made in the last two decades, even though CTCs were first discovered nearly 150 years ago.^57^

The first FDA-approved instrument for CTC analysis, CellSearch, is based on specific molecular recognition, magnetic separation and immunofluorescence.^122^ Using magnetic beads coated with antibodies against CTC surface markers EpCAM, the CellSearch system isolates CTCs upon binding to the beads in blood specimens by applying an external magnetic field.^89^ The cells captured are then fixed and immunostained with fluorescently labeled anti-cytokeratin (CK, an epithelial intermediate filament), a leukocyte specific antibody anti-CD45, and the nuclear stain DAPI, for enumeration by an automated cell imaging and analysis system.^151^ CTCs are recognized as low eccentricity, diameter greater than 5 *μ*m, a visible nucleus, CK-positive staining, and CD45-negative staining. The CellSearch system made possible, for the first time, the isolation and detection of CTCs in a standardized and highly reproducible fashion within a clinical setting.^48^ It has currently become the gold standard to detect CTCs in patients with metastatic breast, prostate and colorectal cancer to guide treatment decisions.^58^ Cristofanilli *et al.* convincingly demonstrated that the survival of metastatic breast cancer patients correlated with CTCs falling below a cutoff of 5 CTCs in 7.5 mL of blood in a multi-institutional study using the CellSearch system.^45^ These results exemplify the value of CTCs for managing treatment groups and monitoring the therapeutic response of metastatic cancer.

While CellSearch represents a breakthrough in CTC isolation and analysis technology both in principle and in clinical applications, there is still great need for more effective, sensitive, and easy-to-use methods to capture and characterize these cells.^73^ A major limitation of CellSearch is that it only captures EpCAM-positive cells.^35^ Our research collaborators have performed approximately 1,500 CellSearch tests on blood specimens from more than 150 metastatic cancer patients. The data from this aggregate of patients are consistent with an earlier study showing that a significant fraction of blood samples lack CTCs.^5^ In addition, CTC outputs from the CellSearch system typically have low yield and purity which may limit further downstream analysis that could be informative for the study of cancer biology and for use in personalized medicine.^48^ Indeed, the system could serve as a platform upon which the body of literature will build a new product which could meet all clinical requirements.^151^

**Figure 2:**
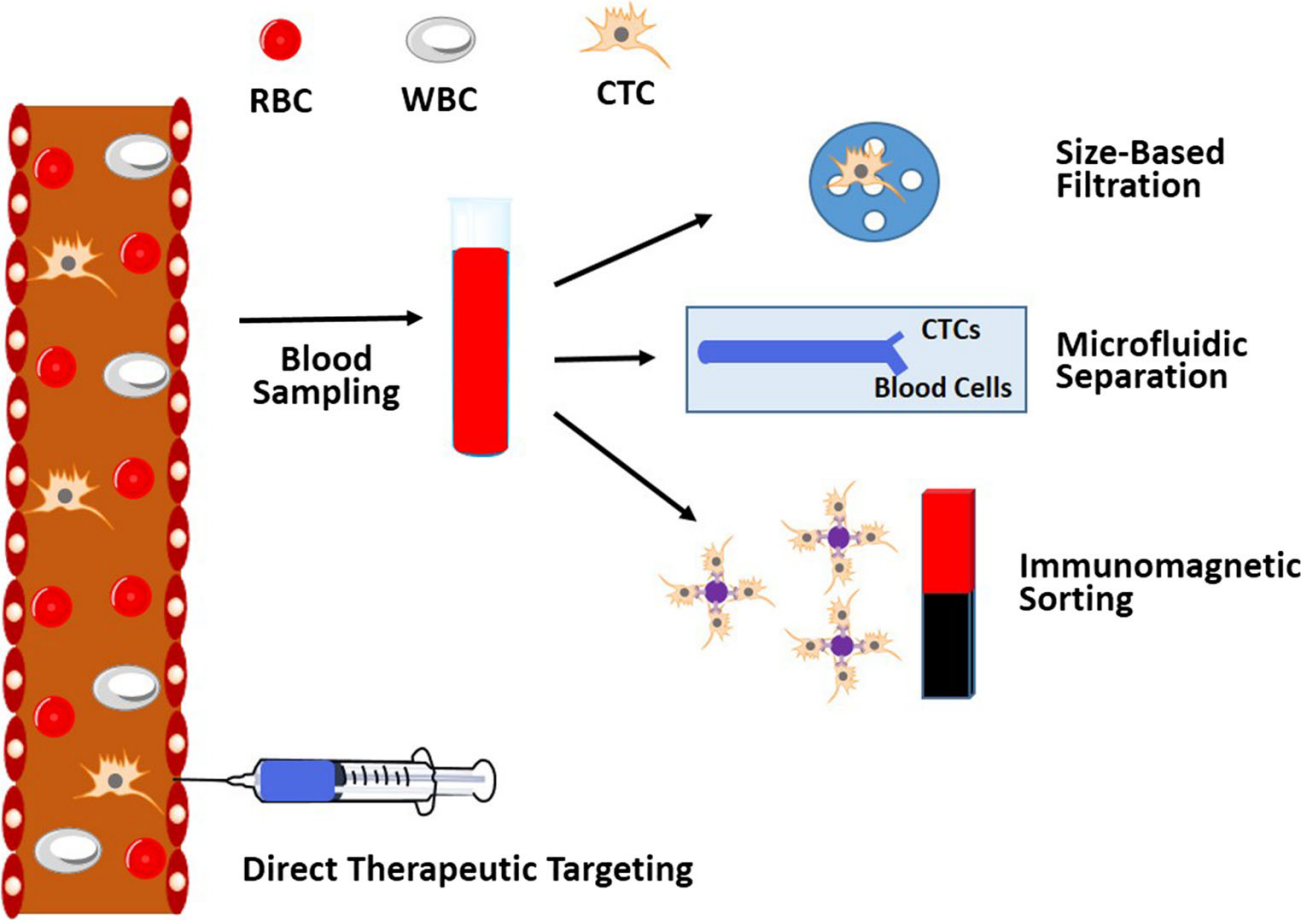
Schematic of various CTC isolation and detection techniques.

The promise that CTC characterization could reveal tumor information at the molecular level has continued to prompt intensive efforts focused on the development of high-performance CTC capture methods since the success of the CellSearch system (Fig. 2).^7^ These include methodologies that isolate CTCs based on molecular recognition, size or other physical properties. For example, the Mag Sweeper uses a slowly rotating magnetic stir bar coated with anti-EpCAM to capture CTCs from whole blood samples, thus improving throughput due to the elimination of the fractionation step.^115^ Many microfluidic approaches are being developed, such as a high-throughput inertial focusing chip that captures magnetically labeled rare CTCs from the blood and enables subsequent RNA-based single-cell characterization.^25^ It’s challenging for standard flow cytometry to detect CTCs due to the relative scarcity of these cells.^80^ One approach to overcome this is to image streaks, by adjusting exposure time to allow very rare cells to be detected and separated.^60^ Label-free methods to detect CTCs from patient samples are also under development. Deformability cytometry is a technique that recognizes CTCs based on their altered mechanical properties, measured as they pass through microfluidic channels under compressive and shear forces.^73^ Alternate approaches to CTC isolation are currently in development, based on the generally held observation that CTCs are larger on average than leukocytes, by processing cell samples through a filter with highly defined pore sizes. Although still in experimental development, these examples represent some of the many exciting new techniques for detecting CTCs.

To date, there is still no FDA approved drugs that directly target metastasis. The molecular characterization of CTCs may offer opportunities for therapeutic targeting of CTCs, which represents a new strategy for the prevention of metastasis. It is an intriguing idea to capture CTCs from a patient sample and then profile these CTCs to decide on the best treatment options. One of the earliest demonstrations of such an approach was the work of Nagrath et al. who achieved highly efficient separation of CTCs from peripheral whole blood samples using an affinity-based microfluidic chip consisting of EpCAM antibody-coated microposts. Owing to the high sensitivity and specificity for CTC isolation, the device was able to measure temporal changes in CTC numbers in patient blood samples following systemic treatment, which was found to correlate well with the clinical course of disease, supporting its potential application in monitoring response to anti-cancer therapy.^107^ Recently, our lab advanced this strategy for screening treatment efficacy of multiple drugs at varying concentrations simultaneously using CTC count as a predictive marker for drug susceptibility.^69^ Cellular therapy has recently emerged as a promising approach for cancer treatment. Tumor infiltrating lymphocytes or genetically engineered T cells have been tested with some success in metastatic cancer patients.^121^ PROVENGE (sipuleucel-T) is the first active cellular therapy approved by the FDA for the treatment of prostate cancer. PROVENGE is implemented by collecting a patient’s own immune cells and then exposing them to tumor-associated antigen to stimulate and direct them to attack prostate cancer.^146^ Following this process, the activated immune cells are then returned to the patient to treat the prostate cancer. PROVENGE is the first autologous anti-cancer cell therapy shown to enhance survival rate.^48^

For the design of new cancer therapies with CTCs as targets, one of the more ambitious approaches is to neutralize CTCs directly in the circulation. This is compelling considering that 90% of cancer deaths are caused by metastasis. The prevention of cancer cell dissemination would thus represent a powerful therapeutic strategy.

In a recent market report, more than 100 companies were cited as providing CTC related products and services.^7^ However, their operating principles and outputs are often at odds with the heterogeneous nature and complex properties of the cells of interest.^151^ Each of these capture and detection methods has their advantages, and the disadvantages associated with the inherent properties of CTCs, as well as the challenging goal of capture and characterization. To account for this, engineering efforts may rely more on disciplines such as nanomaterials to exploit the wealth of technology already established.

During the past two decades, the development of nanotechnology has yielded a variety of new nanoscale materials, including metal, metal oxide, semiconductor and polymeric nanomaterials with a wide range of applications in areas such as medicine, catalysis, electronics, and energy conversion and storage.^11^ The enthusiasm for nanomaterials is based on their exceptional structural and functional properties that are typically different from either bulk materials or discrete molecules due to nanoscale effects. For example, gold nanoparticles (Au NPs) with near-infrared absorption show about one million times higher absorption coefficient than organic dye molecules, making them highly efficient photothermal agents for photoacoustic imaging and tumor ablation. For CTC capture and targeting, nanomaterials have begun to play an increasingly important role.

In recent years, nanomaterials have been intensively investigated for delivering therapeutic agents into solid tumors. The high permeability of tumor vasculature and the lack of proper lymphatic drainage result in the so-called enhanced permeability and retention (EPR) effect in the tumor microenvironment, which is believed to facilitate tumor delivery of nanoparticles and macromolecules that have the ability to circulate with a sufficiently long half-life.^135^ However, nanoparticles must overcome multiple biological barriers before successfully reaching their intended disease sites. The tumor vasculature is highly heterogeneous in distribution and large areas of tumors may be poorly perfused. Impaired lymphatic drainage in tumors contributes to increased interstitial fluid pressure, adding a significant barrier to delivery.^15^ High tumor cell density and dense tumor stroma can further thwart the movement of drugs within tumors. In particular, for delivery to the central nervous system (CNS), the blood–brain barrier (BBB) restricts the diffusion of large or hydrophilic molecules into the cerebrospinal fluid and is a major obstacle for treatment of most CNS and brain disorders.^15^ So far, few nanoparticle technologies have made their way to clinical trials despite an increasingly large number of publications in this area.^21^

All of these barriers, however, are less of a concern when nanomaterials are used to target CTCs in the circulation. Nanomaterials linked with CTC-specific targeting ligands can recognize CTCs with high specificity, allowing for the isolation, detection, characterization, and even direct neutralization using the functional properties of the nanomaterials.^11^ In addition, the large surface-to-volume ratio of nanomaterials offers highly efficient cellular binding in the complex blood and lymph milieu. Furthermore, nanomaterials can be readily manipulated to allow for multiplexed detection and targeting, which are well suited to address the heterogeneous properties of CTCs. CTC detection *ex vivo* has advantages including post-capture analysis and minimal risk of toxicity of the CTC-capturing nanoparticles to the patients. However, a more efficient CTC-capturing nanoparticle could capture extremely rare and heterogeneous CTCs *in vivo*, while maintaining the viability of the captured CTCs, to prevent potentially false negative signals and enable subsequent CTC culture.

This review will focus on using nanomaterials for the capture and therapeutic targeting of CTCs. We systematically categorize nanomaterials, such as liposomes, polymeric nanomaterials, magnetic nanoparticles, gold nanoparticles, quantum dots, and graphenes/graphene oxides, recently developed in the CTC field. Microfluidic devices have become one of the mainstream platforms for CTC enrichment and detection due to many advantages including miniaturization, portability, cost-effectiveness and the ability of online isolation/detection and single cell analysis. Readers are also referred to more specialized reviews and book chapters on related topics.^78^,^87^

## Liposomes

First reported in the 1960s, and soon recognized as a potential drug delivery system, the liposome has since become the most common and well-investigated nanocarrier for research and clinical applications in the field of medicine.^16^,^49^ More than five decades of research have shown their solid benefits to the medical industry.^134^ Several small molecule drugs and genes which were previously deemed less than useful due to issues of stability, solubility, and nonspecific toxicity can now be delivered to their intended sites in the body with the help of liposomes.^32^,^111^

Liposomes are spherical vesicles composed mainly of phospholipids, which form at least one lipid bilayer and an aqueous core within the bilayers.^16^ Ampiphilic in nature, the phospholipids assemble into polar shells in aqueous solutions due to the hydrophobicity of the acyl chains when surrounded by an aqueous medium.^83^ This is a thermodynamically favorable structure further stabilized by hydrogen bonding, van der Waals forces, and electrostatic interactions.^2^,^46^,^125^ Owing to the presence of an aqueous core and a lipid bilayer, liposomes can encapsulate both hydrophilic and lipophilic molecules. The solubility and the *in vivo* fate of the incorporated molecules thus become dependent on the liposomes employed. Advantages of liposomes include improved solubility of the encapsulated drugs, prevention of degradation during storage, reduction of the non-specific side effects and toxicity and improved efficacy and therapeutic index, and versatility when modified with surface ligands for specific targeting.^126^ Thanks to these properties, more than a dozen liposomal formulations have been approved for clinical use, including Myocet and Doxil, with many others currently undergoing pre-clinical development and clinical trials.^9^,^14^

Generally, liposome composition includes natural and/or synthetic phospholipids such as phophatidylcholine (PC), phosphatidylethanolamine (PE), phosphatidylserine, and phosphatidylglycerol. PC and PE constitute the two major structural components of most biological membranes.^138^ Liposome bilayers usually also contain other constituents such as cholesterol and hydrophilic polymer-conjugated lipids. Cholesterol has been largely included to improve the bilayer feature of the liposomes. It improves the membrane fluidity and bilayer stability while reducing the permeability of water soluble molecules through the membrane.^83^ It is a significant step in the development of long-circulating liposomes to include a lipid conjugated polymer, typically poly-(ethylene glycol) (PEG) in liposome compositions.^111^ PEG provides steric hindrance around the liposome surface, preventing access and binding of blood plasma opsonins in the circulation and extending blood circulation time. This technology has yielded a large number of liposome formulations encapsulating therapeutic molecules, with high target efficiency and activity. Furthermore, antibodies or ligands can be conjugated with PEG by modification of the distal terminal group of PEG for active targeting.^54^

There are four classical methods of liposome manufacture: hydration of a thin lipid film, reverse-phase evaporation, solvent injection, and detergent dialysis.^17^,^64^ The difference between these methods is the procedure by which lipids are dried down from organic solvents and then redispersed in aqueous media. These steps are performed individually or are mostly combined.^83^ Here, we only introduce the most widely used method, hydration of a thin lipid film. This is the original method which was initially used for liposome preparation.^36^ A mixture of lipids is first dispersed in organic solvent. Then, the organic solvent is removed by means of evaporation using inert gas flow, vacuum or a rotary evaporator.^111^ Finally, the dry lipid film or cake is hydrated by adding an aqueous buffer solution under agitation at a temperature above the lipid transition temperature. This method is relatively easy to carry out. However, dispersed lipids in aqueous buffer yield a population of multilamellar liposomes, heterogeneous in both size and shape (1–5 µm diameter). Thus, liposome size reduction techniques, such as extrusion through polycarbonate filters, are then needed to produce a smaller and more uniformly sized population of vesicles.

A notable new technique to prepare liposomes with a very narrow size distribution was demonstrated by Jahn and coworkers.^20^,^72^ Liposomes were generated by injecting the lipid phase and the water phase into the microchannel of a hydrodynamic focusing platform. Microfluidic flow is generally laminar due to the microscale channel dimensions and relatively slow flow rates. When the two phases are injected *via* equally sized microchannels, well-defined mixing is then obtained by interfacial diffusion, yielding liposomes with a narrow size distribution. The average size of the liposomes was mainly controlled by varying the flow rate of the two phases.

To assess liposome quality and to obtain a quantitative comparison between different batches of liposomes, several parameters should be measured. For biomedical applications, the main characteristics include the average size and dispersity; encapsulation efficiency; the phospholipid to drug ratio, and lamellarity.^83^ Other commonly monitored parameters include surface charge through zeta potential measurement, phase transitions through differential scanning calorimetry, and quantification of residual solvents through gas chromatography.

Similar to other types of nanomaterials, the average size and size distribution of liposomes are important parameters especially when the liposomes are intended for therapeutic applications. Several techniques are available for assessing nanoscale liposome size and size distribution, which include static or dynamic light scattering, microscopy techniques,^123^ size-exclusion chromatography (SEC),^124^ and field-flow fractionation.^71^

Three general protocols have been established to prepare ligand-bearing PEGylated liposomes.^93^ In the first protocol, PEG-lipids with a reactive end group are incorporated into the liposomes and then conjugated to specific ligands such as monoclonal antibodies after the liposomes are formed.^6^ However, it is possible that some of the reactive end-groups on the liposome surface can cause crosslinking through multiple linkages with a single protein molecule. Thus, sometimes quenching of the unreacted end groups is required. In the second protocol, the ligand-PEG-lipid conjugates are synthesized first and then mixed with other liposome-forming components. The drawback of this approach is that some of the conjugates may not be available for interaction with the target due to the inward orientation to the aqueous core of the liposomes.^126^ The third protocol, which is termed the “post-insertion” technique, involves co-incubation of ligand-PEG-lipid conjugates with preformed plain or PEGylated liposomes.^102^ The advantage of this approach is that all of the ligand moieties are positioned on the outer surface of the liposomes. High-insertion efficiencies can sometimes be obtained when co-incubation conditions (temperature and duration) are optimized. If insertion is performed at elevated temperatures to incorporate into high transition temperature liposomes, protein ligand denaturation can occur. Similar insertion efficiencies might be obtained by changing the incubation to 37°C overnight.

Since 2010, our laboratory has been focused on developing nanomaterials, particularly liposomes, to combat cancer by interacting with, and neutralizing CTCs in the circulation.^48^,^66^–^68^,^79^,^86^,^96^,^99^ The preferred therapeutic is tumor necrosis factor (TNF) apoptosis-inducing ligand (TRAIL), which has shown promise in treating CTCs to reduce the likelihood of metastasis.^143^ TRAIL is a particularly appropriate therapeutic for this delivery method due to its capability to preferentially induce apoptosis in cancer cells, with minimal toxic side effects to most normal cells. In 2012 our group demonstrated, for the first time, that fluid shear stress could sensitize certain human cancer cells to TRAIL-induced apoptosis.^97^ In that study, human colon cancer COLO 205 and prostate cancer PC-3 cells were treated with TRAIL and then exposed to fluid shear stress in a cone and-plate viscometer. Compared to those under static conditions, the two cell lines were induced into a significantly larger decrease in cell viability and more than doubled the amount of apoptotic cells. This shear-induced sensitization to TRAIL-mediated apoptosis was found to be force- and time-dependent. In contrast, the shear treatment did not alter TRAIL-mediated cancer cell necrosis. A general caspase inhibitor blocked the sensitization response while a negative control inhibitor did not, indicating that the shear-induced sensitization to TRAIL was caspase-dependent. It was also found that COLO 205 surface expression of death receptors DR4 and DR5 was not altered by fluid shear stress treatment, which excluded the possibility that sensitization to TRAIL-induced apoptosis was caused by shear-induced changes in receptor expression. Death receptors are known to trimerize and recruit adaptor proteins upon binding to TRAIL to form a signaling complex required for TRAIL-induced apoptosis.^131^ It is possible that a combination of fluid shear stress treatment along with TRAIL binding, rather than fluid shear stress alone, can give rise to changes in death receptor trimerization and signaling. Mechanical shear forces may prompt death receptor trimerization in the presence of TRAIL, leading to the formation of signaling complexes for TRAIL-induced apoptosis. The effects of fluid shear stress on mechanosensing death receptors on the cancer cell surface, along with their signaling pathways, can reveal new strategies for treating CTCs and reducing the occurrence of metastasis.

**Figure 3:**
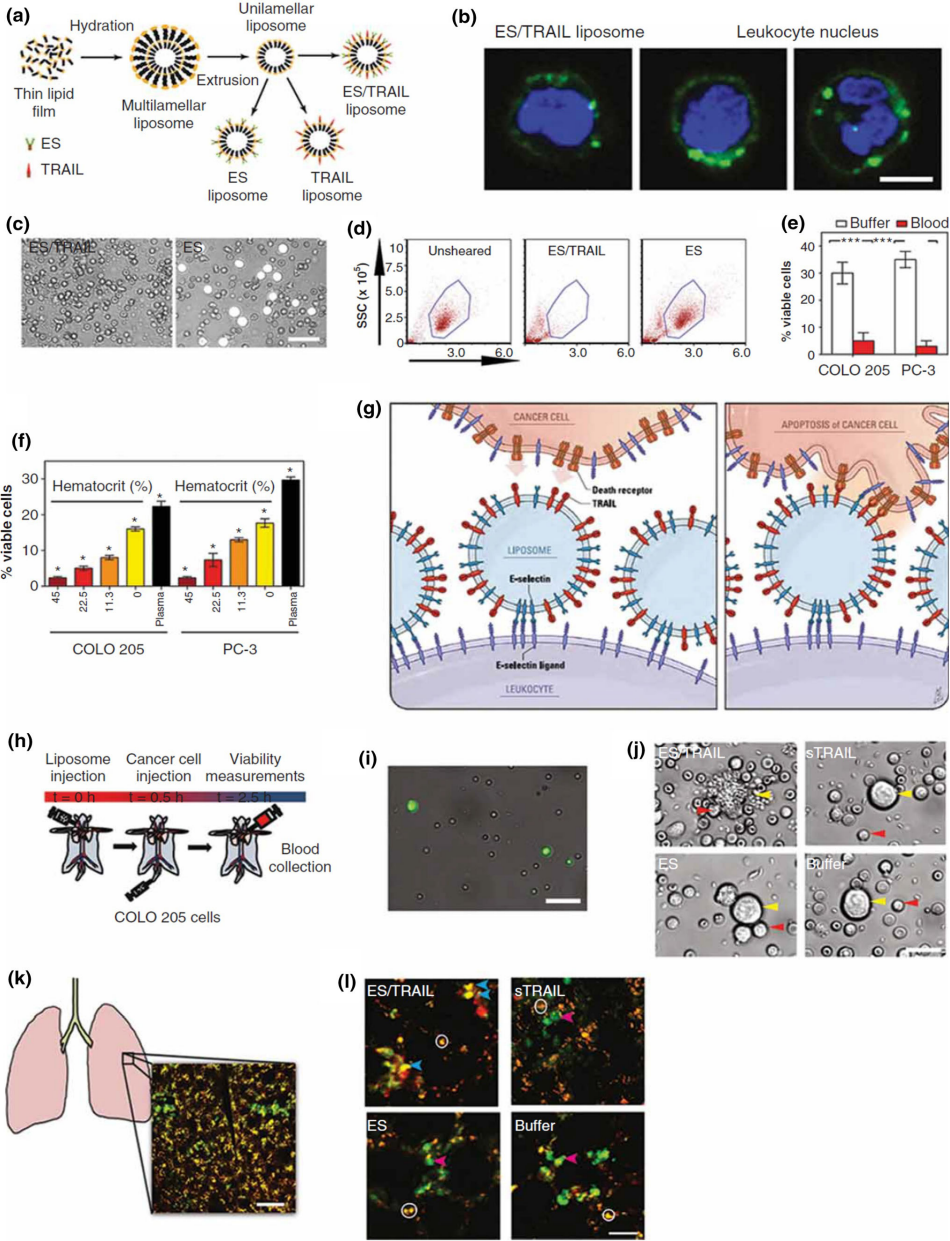
Direct targeting and killing of CTCs using TRAIL/ES liposomes in the blood circulation. (a) Scheme of liposome synthesis. (b) Confocal images of liposomes (green) binding to human leukocytes (blue, cell nuclei). Representative images (c) and flow cytometry (d) of COLO 205 cells (white) with blood cells isolated from blood after liposome treatment under shear flow. (e) Comparison of viability of COLO 205 and PC-3 cells after ES/TRAIL liposome treatment in buffer vs. blood. (f) Viability of COLO 205 and PC-3 cells after treatment with ES/TRAIL liposomes in blood with different percentages of normal hematocrit. (g) Proposed mechanism to explain decoration of leukocytes with liposomes (left), which then contact CTCs and activate the death receptor (right). (h) Schematic of *in vivo* experiment. (i) Leukocytes bound with fluorescent ES/TRAIL liposomes (green) isolated from mouse circulation 2.5 h post-treatment. (Scale bar, 50 *μ*m.) (j) Representative micrographs of COLO 205 cells isolated from the circulation in mice treated with the indicated formulations. (Scale bar, 20 *μ*m.) (k) Schematic of mouse lung with example two-photon excited fluorescence (2PEF) image showing Hoechst-labeled COLO 205 cells (green) are arrested in lung tissue (visible by autofluorescence, yellow). (Scale bar, 80 *μ*m.) (l) The 2PEF images of Hoescht-labeled COLO 205 cells (green) with Alexa Fluor 568-labeled Annexin-V apoptosis probe (red) from the indicated treatment groups. Red arrows point to apoptotic COLO 205 cells (red and green colocalized), blue arrows to non-apoptotic COLO 205 cells (green), and white circles mark autofluorescence from lung tissue. (Scale bar, 30 *μ*m.).

Following the above discovery, we developed liposome-based TRAIL therapeutics to target and kill CTCs in the circulation.^100^ The liposomes, composed of egg l-α-lysophosphatidylcholine (Egg PC), egg sphingomyelin (Egg SM), ovine wool cholesterol (Chol), and 1,2-dioleoyl-sn-glycero-3-[(*N*-(5-amino-1-carboxypentyl) iminodiacetic acid) succinyl] (nickel salt) (DOGS NTA-Ni), were prepared using the hydration of thin lipid film method (Fig. 3a). DOGS-NTA-Ni is a lipid conjugated to nickel-nitrilotriacetic acid (Ni-NTA) that allows for the attachment to his-tagged proteins. TRAIL is conjugated on the surface of nanoscale liposomes along with the adhesion receptor E-selectin (ES) which can recognize and bind to the majority of leukocytes. Successful conjugation was confirmed by detecting the change in average lipsome size and zeta potential before and after conjugation, as well as with TRAIL and ES activity assays. Selectins facilitate rapid, force-dependent adhesion to selectin ligands on tumor cells and leukocytes in blood, which then promote TRAIL ligands to come within a reactive distance of death receptors on the cancer cell surface and initiate the signal for cell apoptosis. These bispecific liposomes, following intravenous administration, adhere to their target leukocytes and enable them to present TRAIL on their surface for the purpose of killing CTCs. Metaphorically, the liposome formulation converts circulating leukocytes into “hunters of CTCs”. ES/TRAIL liposomes consisting of a 10% weight ratio of DOGS-Ni-NTA were found to be the most effective at inducing apoptosis in the COLO 205 cell line under static conditions, as determined with Annexin-V apoptosis assay.

When incubated with COLO 205 cells under flow conditions, the ES/TRAIL liposomes adhered to the cell surface and induced apoptotic cancer cell death. In addition to CTCs, leukocytes also possess surface ligands for ES, which are necessary in the inflammatory response and lymphocyte homing to lymphatic tissues. To assess the potential of the ES/TRAIL liposomes to functionalize leukocytes for targeting and killing CTCs, we treated human blood with fluorescently labeled ES/TRAIL liposomes under shear flow in a cone and-plate viscometer. ES/TRAIL liposomes readily bind to all peripheral blood leukocyte subpopulations upon exposure to shear (Fig. 3b). As revealed by Annexin-V assays, the ES/TRAIL liposomes show negligible cytotoxic effects to leukocytes and human endothelial cells under blood flow conditions. To examine whether ES/TRAIL liposomes would effectively target cancer cells in whole blood under flow conditions, we spiked fluorescently labeled colorectal COLO 205 or prostate PC-3 cancer cell lines into human peripheral blood. Surprisingly, ES/TRAIL treatment was even more effective for killing cancer cells in human blood, with less than 5% of the spiked cancer cells remaining after 2 h of treatment (Figs. 3c–3e). Contrary to typical observations that the therapeutic efficacy of most synthetic reagents in the blood decreases due to cellular internalization and nonspecific binding of plasma proteins, the efficacy of ES/TRAIL liposomes was enhanced in human blood, compared to conditions in buffer. Interestingly, the apoptotic effects were found to be hematocrit-dependent, with higher hematocrit significantly decreasing the number of viable cancer cells after ES/TRAIL treatment (Fig. 3f). It has been speculated that ES/TRAIL liposome attachment to the leukocyte surface can enhance their ability to induce cancer cell apoptosis due to the compressive forces between cancer cells and leukocytes under flow. Compressive forces can act to flatten the glycocalyx protection layer around cancer cells, thus allowing TRAIL to come into closer contact to the cancer cell death receptors for apoptotic action.^98^ This approach is intended to neutralize rare CTCs in blood, and the margination of leukocytes and CTCs along the vessel wall results in CTCs becoming surrounded by the circulating leukocytes (Fig. 3g). Thus, individual CTC will essentially become surrounded by both adhesion receptors and therapeutic ligands upon entering the bloodstream when leukocytes in blood have been functionalized with TRAIL/ES liposomes. This represents a unique strategy to target and kill rare CTCs to prevent the development of distant metastases.

To evaluate the *in vivo* efficacy to remove CTCs from the circulation, ES/TRAIL liposomes were injected into immunocompetent mice *via* tail vein before injection of COLO 205 cells (Fig. 3h). Remarkably, negligible viable cancer cells were found still in the peripheral blood after only 2 h treatment compared to controls (Figs. 3i, 3j). An examination of the remaining cancer cells within the mouse vasculature using multiphoton microscopy showed a decreased number of cancer cells in the lungs of treated mice, with the majority of the remaining cancer cells in apoptosis in treated mice but not in the control group (Figs. 3k, 3l).

In addition to the above cited advantages of this strategy, attachment of TRAIL/ES liposomes to the surface of leukocytes in blood is beneficial to increase TRAIL circulation time, by avoiding renal clearance. By focusing the therapeutic effects to within the vascular microenvironment, TRAIL dosages can be significantly reduced, as the dosages used in this study were approximately two orders of magnitude lower than the dosages used in previous human clinical trials of TRAIL protein. Representing an important first step in targeting CTCs in the bloodstream, the “unnatural killer cells” approach can potentially be adopted as a preventative measure upon diagnosis of highly metastatic hematogenous cancers that originate from epithelial tissues including breast, prostate, and lung.

**Figure 4:**
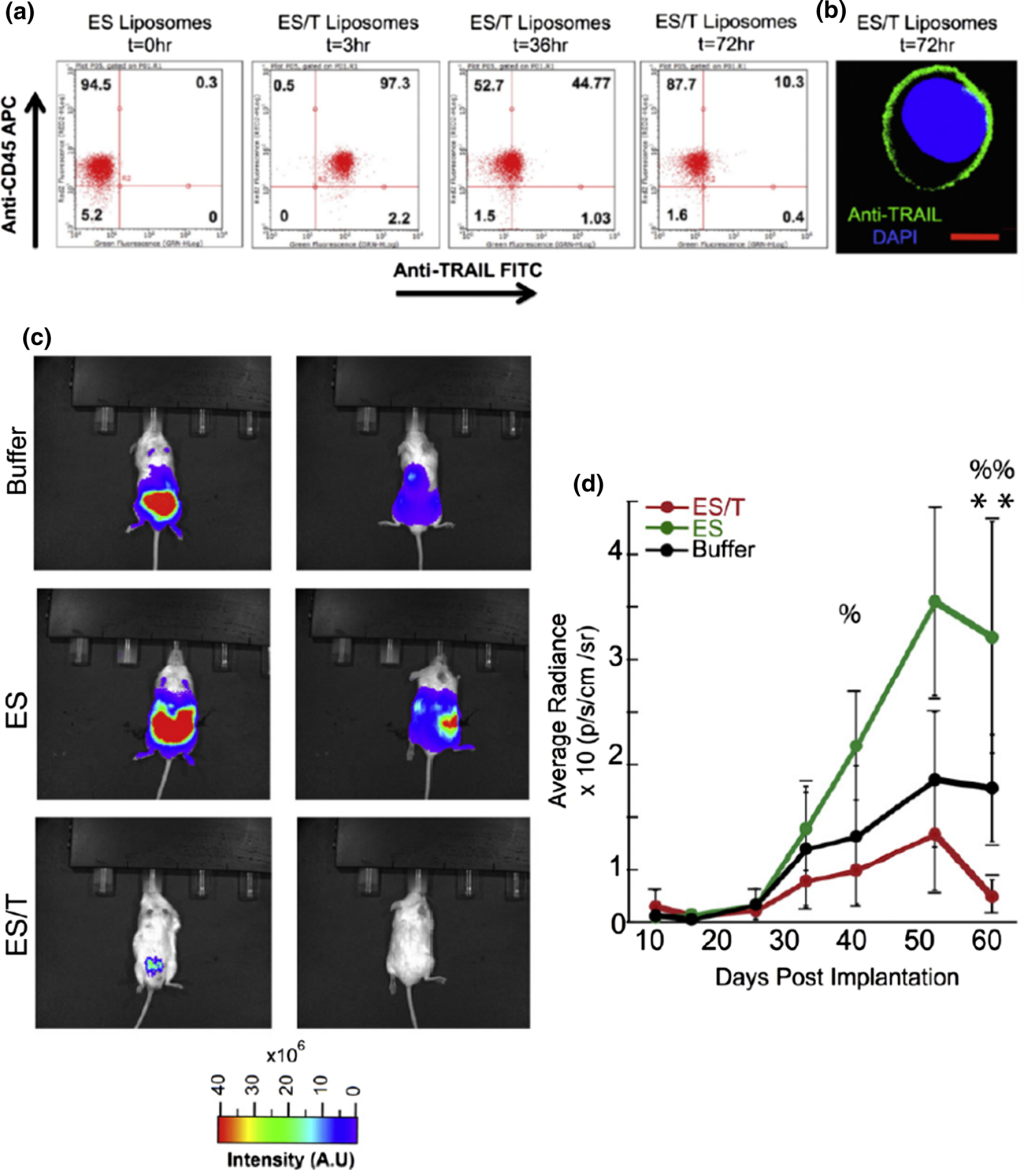
Evaluation of pharmacokinetics and efficacy of TRAIL-coated leukocytes. (a) Flow cytometry analysis of circulating mouse leukocytes staining positive for surface bound human TRAIL protein. (b) Confocal image of a mouse leukocyte presenting human TRAIL on its surface (Scale bar = 10 *μ*m). (c) Whole animal BLI of the ventral (left) and dorsal (right) sides of representative mice from the indicated groups at the end of the treatment (week 9). (d) Fluorescent quantification of the burden of the primary tumor. (ES vs. ES/TRAIL: % *p* < 0.05, %% *p* < 0.01; Buffer vs. ES/TRAIL: ** *p* < 0.05.) (Adapted with permission from Ref. 145. Copyright © 2015 Elsevier B.V.)

In another project, we evaluated the efficacy of the same TRAIL/ES liposomes to prevent the spontaneous formation and growth of metastatic tumors in an orthotopic xenograft model of prostate cancer.^145^ Prostate cancer is known for having poor prognosis and limited treatment options once it has progressed from its local to metastatic form. We first performed pharmacokinetic studies to determine the half-life of TRAIL/ES liposomes in the circulation to estimate an appropriate timescale for ES-TRAIL treatment. Leukocytes were isolated from mouse blood following injection of the liposome treatment, stained with leukocyte and TRAIL specific, fluorescently labeled antibodies. It was found that nearly 100% of leukocytes showed human TRAIL on their surface within 30 min, and the percentage decreased to 10% at 72 h (Figs. 4a, 4b). The half-life of the formulation was estimated to be ~30 h, significantly longer than that of conventional, non-targeted stealth liposomes. In treatment experiments, TRAIL/ES liposomes were injected every 72 h with the first injection performed one week prior to the time when CTC populations were first detectable in the circulation. Six weeks later, metastatic tumors spread widely throughout the abdominal cavity in the two control groups as revealed by whole animal BLI while no macroscopic metastases were visible in the ES/TRAIL treatment group. Remarkably, the treatment also significantly reduced the growth rate of the primary tumor, with no BLI signal detectable in treated mice when imaged from the dorsal side (Fig. 4c, 4d). At the end of the treatment, CTC count in the blood of buffer-treated mice vs. TRAIL/ES liposome-treated mice showed a difference of around 94%. Tumor growth in distant organs was assayed to determine whether TRAIL/ES liposomes were effective in reducing metastases. In the control groups, metastatic tumors were found in the lungs and liver, kidneys and spleen with none found in TRAIL/ES liposome treated mice. It is important to note that the dosage of TRAIL used in this TRAIL/ES liposome formulation is about 1.0% of the concentrations that have been well tolerated in previous animal and human trials with soluble TRAIL protein, and in fact we have not observed any evidence of toxicity. To summarize, for the first time, it was shown that such an approach can be used to prevent the spontaneous formation and growth of metastatic tumors in an orthotopic xenograft model of prostate cancer, by greatly reducing the number of circulating tumor cells. It was concluded that the “hitchhiking” TRAIL protein could be an effective tool to directly target CTCs for the prevention of prostate cancer metastasis, and potentially other cancers that spread *via* the blood circulation.

Another liposome formulation of TRAIL that we have developed for the prevention of metastasis is to target CTCs in the lymph nodes.^24^ Despite being the home of many immune cells that are capable of responding to tumor antigens, the lymph nodes are the first organ of metastasis in several types of carcinoma and melanoma. The main reason for this is that cancer cells can evade the host immune response. Sentinel lymph nodes (SLN) are the first set of lymph nodes that primary tumor cells encounter during metastasis. The immune function of SLN, to eliminate micrometastatic lesions, may be suppressed as suggested by their morphological and functional variation in cancer patients. Augmenting the host immune response can reverse the immune suppression in SLN and thus represents a promising strategy for prevention of metastases. Instead of using the entire peripheral blood leukocyte population in our liposome formulation of TRAIL to target CTCs in the blood, we used natural killer (NK) cells to target CTCs specifically in the lymph nodes. NK cells are cytotoxic lymphocytes capable of delivering lethal bursts of toxins that activate apoptotic pathways in cancer cells. Indeed, it is NK cells that kill most of the CTCs in the circulation. However, in cancer patients, NK cells are found to show several abnormalities such as reduced count, decreased cytotoxicity, and poor tumor infiltrating capacity. Most of these abnormalities can be exaggerated in patients due to immunosuppression by chemotherapy. We tethered TRAIL onto the surface of NK cells *via* liposomes to target CTCs in the lymph nodes.

We used a formulation of hydro soy PC L-α-phosphatidylcholine (HSPC), cholesterol, 1,2-distearoyl-sn-glycero-3-phosphoethanolamine-*N*-[methoxy (polyethyleneglycol)-2000] (DSPE-PEG) and 1,2-distearoylsn-glycero-3-phosphoethanolamine-*N*-[maleimide (polyethyleneglycol)-2000] (DSPE-PEG-Mal). The maleimide group at the distal end of PEG allows conjugation of proteins after thiolation. Liposomes were composed of HSPC:cholesterol:DSPE-PEG:DSPE-PEG-Mal at a molar ratio of 2:1:0.08:0.02. TRAIL and an antibody to CD57 (anti-CD57), a marker for terminal differentiation in T-cells, were thiolated and conjugated with the preformed liposomes for testing with human NK cells.

The formulation was first tested using a cell culture platform for mimicking lymph node micrometastases, termed microbubbles (MBs) formed in polydimethylsiloxane (PDMS) from a microfabricated silicon wafer. The MB had spherical geometry to mimic the deep cortical unit of lymph nodes. Cancer cell lines cultured in microbubbles form small micrometastasis-like spheroids. NK cells isolated from whole blood were modified by incubation with the liposomes. Flow cytometry analysis revealed that anti-CD57 on the liposome surface mediates conjugation of the liposomes to NK cells. Cancer cells including MDA-MB-231, COLO 205 and LNCaP cells, progressively became apoptotic when cultured with liposome-modified NK cells in MBs. Since lymph node metastases are common in several types of cancer, the NK cell engineering approach may be useful for developing cancer therapies to sweep lymph node micrometastases *in vivo*.

**Figure 5:**
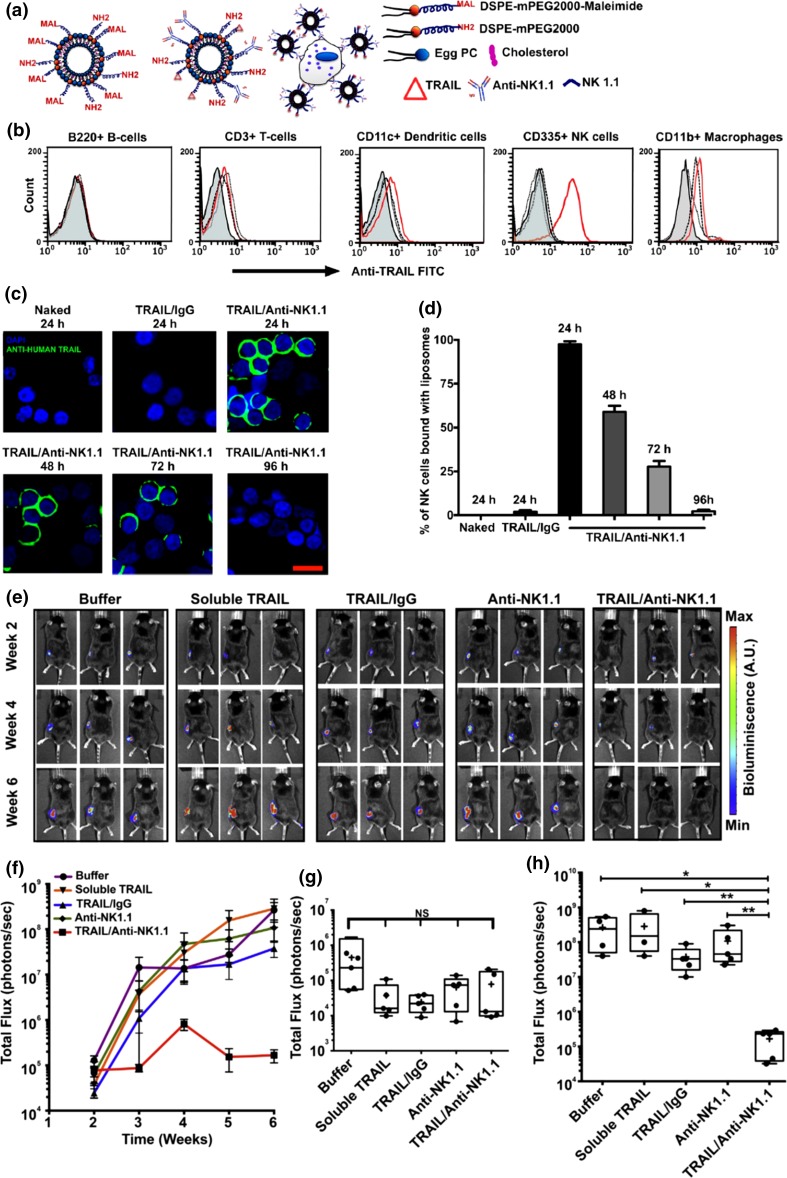
Pharmacokinetics of TRAIL/Anti-NK1.1 liposomes and metastatic burden in the tumor draining inguinal lymph nodes. (a) Schematic of liposome formulation. (b) Flow cytometric analysis of the interaction of liposomes with the indicated type(s) of cells within the lymph nodes 24 h post-injection. The mice were injected with buffer (filled), naked liposomes (dash), TRAIL/IgG liposomes (dot) or TRAIL/Anti-NK1.1 liposomes (red). (c) Representative fluorescent confocal micrographs of NK cells isolated from the inguinal lymph nodes of mice subcutaneously injected with the indicated formulations. (Blue, nuclear stain DAPI; Green, anti-human TRAIL; Scale bar, 20 µm.) (d) Numerical quantification of the percentage of NK cells functionalized with the indicated types of liposomes. The bars represent the mean and standard deviation. (e) Sequential bioluminescence imaging shows the growth of inguinal lymph node metastases in mice following the indicated treatments. (f) Fluorescent quantification of the tumor burden in the inguinal lymph nodes with time. Box and whisker plot comparing total flux from the inginal lymph nodes in mice from different treatment groups at week 2 (g) and week 6 (h). (NS, not significant; * *p* < 0.05; ** *p* < 0.01.) (Adapted with permission from Ref. 24. Copyright © 2015 Elsevier Ltd.)

We then evaluated if TRAIL liposomes targeted to NK cells could effectively prevent the metastasis of a primary tumor to the tumor-draining lymph nodes in a subcutaneous human xenograft tumor model. In the liposome formulation for this study, the antibody to target NK cells was anti-NK1.1 antibody which was isolated by mouse hybridoma cell line (Fig. 5a). For pharmacokinetic study, TRAIL/Anti-NK1.1 liposomes and TRAIL/IgG liposomes as a control were injected in mice on both the left and the right abdominal flanks. Lymph nodes were harvested and immune cells were isolated for TRAIL quantification using fluorescently labeled antibodies. Adhesion of TRAIL/Anti-NK1.1 liposomes was observed for NK cells but not for other immune cells, thus confirming the specific interaction of liposomes with NK cells in the inguinal lymph nodes (Fig. 5b). At 72 h post-injection, ~28% of NK cells were still bound with liposomes (Figs. 5c, 5d). To establish the *in vivo* tumor model, SW620 cells were injected subcutaneously into the lower left abdominal flank for spontaneous metastasis to the inguinal lymph nodes. Liposomes were injected subcutaneously adjacent to the primary tumor 2 weeks after tumor implantation. The mice were monitored weekly using BLI for the growth of the primary tumor and the tumor burden in the tumor draining inguinal lymph nodes (Fig. 5e). The control groups (buffer, soluble TRAIL, TRAIL/IgG liposomes and Anti-NK1.1 liposomes) developed increased tumor burden in the skin-draining inguinal lymph nodes while the TRAIL/Anti-NK1.1 treatment group showed a dramatic reduction in the ability of the primary tumor to metastasize to the inguinal lymph nodes (Figs. 5f–5h). Importantly, no change in NK cell activity or toxicity to the local lymph node tissue was detected. To summarize, it was shown that liposomes conjugated with both TRAIL and NK cell antibody were carried to the tumor-draining inguinal lymph nodes and prevented the lymphatic spread of a subcutaneous tumor in mice. By conjugating with NK cells, the circulation time of liposomal TRAIL was greatly enhanced. It was concluded that the TRAIL/anti-NK liposomes are effective in killing CTCs within the tumor draining lymph nodes to prevent the lymphatic spread of cancer.

## Polymeric Nanomaterials

Polymers constituted the earliest drug delivery systems introduced in the 1970s.^113^ Today, polymeric materials still represent a large category of nanomaterials for the delivery of cancer drugs, primarily because they are typically biocompatible and biodegradable and researchers can readily control their chemical and physical properties by molecular synthesis.^28^,^74^,^110^ Recent advances in polymeric nanomaterials have allowed researchers to design biomedical applications with versatile functions.^103^,^108^

Linear amphiphilic block copolymers form nanoscale core–shell particles, called “micelles”, *via* a self-assembly process in an aqueous environment, with a hydrophobic block forming the core to minimize aqueous exposure, and a hydrophilic block forming the shell to stabilize the core.^43^ The shell of the micelles provides steric protection, while the core allows for loading of hydrophobic small-molecule drugs. Hydrophilic drugs including macromolecules such as nucleic acids and proteins can also be loaded *via* electrostatic attraction or chemical conjugation. Poly (lactic-co-glycolic acid) (PLGA) is one of the most successfully used polymers for biomedical applications because its hydrolysis leads to metabolite monomers, lactic acid and glycolic acid, and can be utilized to deliver a variety of cancer drugs.^137^ Poly(lactic acid) (PLA) and chitosan based polymers have also been widely used to develop polymeric micelles due to their biodegradable and biocompatible properties. Similar to PLGA, PLA possess low toxicity, with an additional slightly negative surface charge.^140^ Chitosan, a polysaccharide with structural characteristics similar to glycosaminoglycans, has been widely utilized to serve as a carrier of hydrophilic drugs.^75^ Encapsulation of doxorubicin-dextran conjugates within chitosan nanoparticles has been shown to reduce the nonspecific side effects of doxorubicin while enhancing the therapeutic index in the treatment of solid tumors *in vivo*.^13^

The release of therapeutic agents from polymeric micelles traditionally has been largely diffusion-controlled.^76^ Currently, however, modern research is aimed at investigating stimuli-responsive polymers the release from which can be triggered by various physical stimuli, such as light, temperature, pH, and mechanical stress.^119^ One major concern with utilizing polymeric micelles is that most of them demonstrate a wide size distribution, as indicated by a high polydispersity index.^104^,^154^ However, certain methods can now produce polymeric micelles with monodisperse size distributions.^77^

Most polymers investigated for CTC capture are indeed incorporated into microfluidic chips or a surface without forming micelles.^3^,^90^ Only very few of them formed free micelles to capture CTCs. For example, one group demonstrated success in suppressing tumor metastasis by killing CTCs using a doxorubicin (Dox) loaded biodegradable polymeric micelle.^33^ They synthesized monomethyl poly(ethylene glycol)-poly(*ε*-caprolactone) (mPEG-PCL) diblock copolymer which was then used to prepare doxorubicin-loaded micelles using a pH-induced self-assembly method. The Dox loaded micelles were measured to be ~27 nm and nearly monodisperse with a high Dox encapsulation efficiency. The micelles themselves exhibited minimal cytotoxicity to 4T1 cells even at very high concentration while the Dox loaded micelles exhibited slightly higher cytotoxicity than free Dox with 4T1 cells. In transgenic zebrafish, Dox micelles inhibited tumor growth, prolonged the survival of tumor-bearing zebrafish, and suppressed tumor metastasis by killing CTCs. Improved anti-tumor and anti-metastatic activities were also found in mouse tumor models, where immunofluorescence staining of tumors indicated that Dox micelles induced more apoptosis and showed fewer proliferation-positive cells.

Another type of polymer with potential use for CTC capture is the dendrimer, which is a repetitively branched molecule that adopts a spherical nanoscale three-dimensional morphology.^44^ Dendrimers offer a unique opportunity to precisely mediate the multivalent binding effect obtained from their well-defined structure and a high density of surface functional groups. The CTC binding ability of a capture surface can be enhanced through dendrimer-mediated multivalent binding effects, which can significantly improve the sensitivity and selectivity of the surfaces for CTC capture and detection.^105^ One group reported a CTC detection platform for the capture of tumor cells by taking advantage of the PAMAM dendrimer-mediated multivalent binding effect.^105^ They conjugated PAMAM dendrimers to a glass slide surface *via* a PEG linker. The dendrimer-functionalized surface was then incubated with anti-EpCAM, resulting in a CTC specific capture surface. The functionalized surface exhibited dramatically enhanced cell adhesion and binding stability of three different breast cancer cell lines, MDA-MB-361, MCF-7, and MDA-MB-231. Compared to a linear polymer PEG-coated surface, the surface capture of tumor cells on the dendrimer-coated surface was significantly improved.

## Magnetic Nanomaterials

One commonly used strategy for the isolation of CTCs from the circulation is to utilize magnetic nanomaterials that bind to the cells *in vivo* or *in vitro* for separation, by applying an external magnetic field.^4^,^39^ Here, magnetic nanomaterials refer to individually dispersed magnetic nanoparticles (MNPs), or clusters of MNPs assembled in an organic or inorganic matrix.^94^,^149^ MNPs are commonly composed of iron oxide, cobalt ferrite, and chromium dioxide, and show alignment of their magnetic moment in the presence of an external magnetic field.^136^ Iron oxide MNPs that are chemically inert and biocompatible have been most commonly used in CTC isolation.^51^,^118^ Depending on the particle size, the magnetic response of iron oxide MNPs can be ferromagnetic, showing a remnant magnetization after removal of the external magnetic field, or superparamagnetic, showing no remnant magnetization.^84^ Generally, only superparamagnetic nanoparticles (SMNPs) or clusters composed of SMNPs can be used for cell isolation since ferromagnetic nanoparticles are inherently attracted to each other and cannot be stably dispersed in aqueous media. Naked individual SMNP will also become aggregated in aqueous media without appropriate surface protection.^92^ Therefore, it is often necessary to modify the surface of SMNPs by grafting of, or coating with, surfactants, polymers (e.g., PEG, dextran, chitosan, and polypeptides), or hydrophilic inorganic materials (e.g., silica). A protective shell not only helps to solubilize MNPs, but can also be used for further conjugation with other functional groups such as ligands for a wide range of biomedical applications. Magnetic separation using specific ligand-conjugated MNPs or clusters of MNPs is one of the most popular CTC isolation methods.^129^ The separation can be easily manipulated by applying or removing an external magnetic field, and exhibits satisfactory capture efficiency and specificity. Captured cells can be easily collected by disrupting cell-antibody binding using enzymes.

Indeed, the FDA-approved CellSearch system employs 120–200 nm clusters composed of small iron oxide nanoparticles for CTC isolation.^122^ Another commercialized approach that utilizes the magnetic enrichment technique is AdnaTest.^27^ This equipment uses a dual-capture strategy, in which CTCs are captured with a mixture of large microbeads conjugated with one of two different antibodies: a general cancer cell antibody against EpCAM, and the other against a tumor type specific marker such as MUC-1 or HER2 depending on the type of cancer. Subsequent analysis is done with multiplexed RT-PCR to identify tumor-associated mRNAs. Compared with the CellSearch system, AdnaTest expands the isolation step by capturing CTCs with either one of the two antigens on their surface. However, no critical comparison between the two systems has yet been made to show if one outperforms the other for various specific purposes. Distinct from CellSearch and AdnaTest, the commercialized magnetic activated cell sorting (MACS) system entraps CTCs labeled with SMNPs within a magnetized steel wool column.^106^ When the column is removed from the external magnetic field, the trapped cells are freed from the steel wool and collected after elution using an aqueous buffer.

While positive CTC selection is appealing, a drawback of this method is that CTCs that do not express the targeted markers will not be recognized and captured. This problem can be partly addressed by using a negative depletion strategy with magnetic beads.^155^ A general approach for negative selection with magnetic separation is to first lyse RBCs and then use MNPs modified with anti-CD45 antibodies to remove WBCs. As demonstrated by Yang and co-workers, this method can reduce the number of normal blood cells by two orders of magnitude.^150^ It produces a recovery rate of approximately 83% with spiked cancer cell lines. However, due to the great number of normal blood cells, it is indeed very challenging to reduce the number of background cells to a sufficient degree to increase CTC detection efficiency. In addition, the lysis procedure may damage some rare CTCs, producing false negatives.

## Gold Nanomaterials

Nanoscale particles of gold currently attract a great deal of research attention for biomedical applications.^30^,^34^,^62^ Enhanced light absorption and scattering properties of gold nanoparticles (Au NPs) compared to their bulkier counterparts have been employed for detection of CTCs, as the binding between Au NPs and CTCs can be quantitatively measured *via* photoacoustic signals or shifts in surface plasmon resonance (SPR).^12^,^141^ Au NPs with different shapes, such as gold nanospheres, nanoshells, and nanorods, can be prepared and modified with targeting ligands, imaging labels, therapeutic drugs, and other functionalities.^61^,^132^ Au NPs have been hotly pursued for *in vivo* imaging and as *ex vivo* diagnostic sensors due to their ultrahigh sensitivity, throughput, and flexibility. The SPR of Au NPs varies depending on particle size and shape: the narrow range of nanospheres (~520–550 nm); splitting into two modes of nanorods (transverse and longitudinal at 520–550 nm and 650–900 nm, respectively); and near-infrared (NIR)-closing range of nanoshells (850–900 nm).^63^ In particular, due to this unique SPR splitting, gold nanorods have been frequently employed for CTC detection using techniques such as photoacoustic and surface enhanced Raman imaging.^109^,^133^,^139^

CTCs can be detected *in vivo* by injecting Au NPs modified with CTC-specific ligands, enabling* in situ* enumeration of CTCs. Real-time CTC monitoring *in vivo* avoids the necessity of blood sampling, sample preparation, or CTC isolation, and may trigger the phagocytic clearance of CTCs upon binding.^40^,^106^ However, for this appealing strategy to be effective, the Au NPs must overcome the high shear stress of the blood circulation, evade immune responses, and avoid undesired accumulation in organs.^56^,^128^ This method may also result in false positive signals due to the presence of target antigens/markers on normal cells and nonspecific binding of the nanoparticles. PEGylation of Au NPs has been frequently used to address some of these issues by extending their circulation time while diminishing non-specific binding.^19^,^117^ Photoacoustic flow cytometry using Au NPs has been developed in recent years for the detection and ablation of CTCs *in vivo*. This technology functions by applying a laser through the skin into a vessel and detecting the acoustic vibrations that result from the nanoparticle absorption of laser energy.^37^,^40^ One typical application of this technology is for the detection of circulating melanoma cells, taking advantage of their endogenous expression of melanin nanoparticles.^10^,^47^ Increasing the incident laser energy can lead to the generation of heat by plasmonic Au NPs to a sufficient degree that the flowing melanin-containing cells are ablated.^41^

Au NPs functionalized with CTC-specific ligands can be used *ex vivo* either to directly bind to and separate the CTCs from blood samples, or to functionalize a surface to isolate CTCs from blood.^38^,^109^ The advantages of CTC detection *ex vivo* include enabling subsequent cell culture and analysis as well as minimal risk of potential toxicity of the CTC-capturing nanomaterials for patients.

Au NPs can also be functionalized on a nanostructured surface to enable binding with CTCs and non-labeling enumeration. For example, an Au NP-modified chip was fabricated from the silica surface of a master mold using the wet-lithographic method and then coated with a monolayer of 11-mercaptoundecanoic acid to capture CTCs, followed by antibody immobilization.^42^ The antibodies bound to the gold nanostructured surface and binding interactions on the sensor chip surface were successfully quantified in real time by monitoring the phase shift and magnitude of the electroacoustic resonance in the collected signal. Using lower levels of antibody, the gold nanostructured chip successfully detected two different types of cancer cells, whereas a reference surface covered with IgG did not.

## Quantum Dots

The creation and application of fluorescent semiconductor nanocrystals, commonly referred to as Quantum dots (Qdots), is considered a breakthrough in optical biomedical imaging.^95^ After more than a decade of intensive research, Qdots have been demonstrated to be a powerful fluorescence imaging agent for *in vitro* applications.^130^,^148^ Compared to conventional dyes, Qdots exhibit several advantages for biological imaging. The most obvious advantage is that they are exceptionally bright due to much larger extinction coefficients and comparable quantum yields.^18^ This quality provides enhanced sensitivity and earlier detection of biological events. Another major advantage is that they are highly resistant to photobleaching which allows continuous dynamic imaging over minutes to hours.^156^ Qdots also have a broader excitation spectra and a narrower emission spectra.^27^ Qdots provide a unique opportunity to capture CTCs in a quantitative manner. Colloidal Qdots are commonly composed of ZnS, CdS, ZnSe, CdTe, or PbSe.^23^ The solubility issue of Qdots can be solved by surface functionalization with polymeric ligands or a layer of hydrophilic inorganic materials, either through ligand exchange or by additional coating.^59^ Surface-functionalized Qdots have been used in deep-tissue imaging,^8^ fluorescence resonance energy transfer (FRET)-based cellular labeling,^26^ and CTC detection as well.^116^,^120^ Among those applications, *ex vivo* CTC detection using Qdots has the greatest clinical relevance, in view of their strong, stable fluorescence and yet with manifest heavy metal toxicity when used *in vivo*.^38^,^55^

The strong and stable fluorescence emission of Qdots has given rise to the development of Qdot-based fluorescent probes for CTC detection. For instance, Qdots conjugated with aptamer-DNA concatemer that bind to CTCs have been used to detect CCRF-CEM (T lymphoblastoid) cells in the range of ~ 100 cells/mL.^91^ It is desirable to be able to detect multiple surface markers simultaneously on CTCs. This idea has been demonstrated in a proof-of-concept study.^85^ Three different sized Qdots with narrow emission at 525, 565, and 625 nm were modified with antibodies against EpCAM, epidermal growth factor receptor (EGFR), and human epidermal growth factor receptor (HER-2), respectively. A mixture of the functionalized Qdots was used to identify and sort three different breast cancer cell lines, MCF-7, MDA-MB-231, and SK-BR-3 based on their distinct fluorescence emission wavelengths. The capture efficiency and surface marker-dependent sorting accuracy of the cancer cells reached 87.5 and 92.4%, respectively. Importantly, the captured CTCs were released without influencing cellular viability, which is crucial for post-capture analysis.

## Graphenes and Graphene Oxides

Graphenes are a two-dimensional monolayer of sp^2^ hybridized carbon arranged in a hexagonal packed structure.^152^ Graphenes possess numerous extraordinary physicochemical properties, high intrinsic mobility, strong mechanical strength, and excellent electrical conductivity.^144^ With every atom exposed on its surface, graphene also has an ultrahigh specific surface area available for functionalization on both sides.^112^ Thanks to these properties, graphenes and their oxidized form graphene oxides (GO) have been explored for electrical detection of CTCs through incorporation with other materials or in devices.^1^,^53^,^147^

Several CTC capture and imaging applications are based on the fluorescence quenching properties of GO.^29^,^65^,^88^,^153^ The hydroxyl and carboxyl groups of GO allow for chemical conjugation or electrostatic interaction with fluorescent molecules, while its aromatic, sp^2^ domains facilitate π-π stacking and fluorescence quenching. The quenched fluorescence is recovered upon interaction with a target marker on the cell membrane. In this respect, fluorescently labeled DNA and proteins were adsorbed and functionalized on GO for use as optical probes.^142^ Four distinct dye conjugates with four distinct aptamers respectively were used to modify the surface of a microporous (20–40 *μ*m) GO membrane. Multiple types of tumor cells were captured simultaneously on the GO membrane through specific binding of these aptamers to tumor biomarkers, such as HER-2, prostate specific membrane antigen, and carcinoembryonic antigen. The GO membrane achieved 95% capture efficiency with nearly 100% cell viability from whole blood spiked with a mixture of three different types of cancer cells and one normal cell type as a negative control. The captured cells were visualized and differentiated on the GO membrane through multicolor fluorescence imaging after fluorescence recovery.

## Conclusion

The capture and characterization of CTCs from the peripheral blood has yielded results suggesting promising clinical relevance of CTCs, as well as insights into mechanisms of cancer progression. This led to the successful development of CellSearch for CTC numeration, which has been used clinically for over a decade. Furthermore, preliminary results suggest that CTC enumeration and analysis may serve to monitor the antitumor activity of an ongoing therapy. Numerous clinical studies using different CTC capture and detection assays have been conducted for a wide range of distinct tumor types at different disease stages. However, sensitivity and specificity remain the key issues to be addressed in upcoming technologies which are expected to meet higher criteria. Nanomaterials seem promising to fulfill these purposes. While each type of the above-discussed nanomaterials has their advantages and drawbacks for CTC capture and therapeutic targeting (Table 1), many of them can be modified with multiple functionalities and are ideally suited to reduce false negatives, bridge current isolation and detection methods, and enable multiplexed targeting.

**Table 1:** Summary of nanomaterials for CTC capture and therapeutic targeting.

Nanomaterials	Pros	Cons	Examples (references)
Liposomes	Biocompatibility, long blood circulation time	Low stability	24,98,100,145
Polymeric nanomaterials	Biocompatibility and biodegradability, ease for chemical modification	Low stability	33,105
Magnetic nanoparticles/nanoclusters	Biocompatibility, magnetic separation	Magnetic aggregation	27,106,129,150,155
Gold nanoparticles	High stability, laser-controlled simultaneous detection and ablation	Non-biodegradability	10,37,38,41,42,47
Quantum dots	Quantitative detection with high sensitivity	Toxicity	85,91
Graphene/graphene oxides	Ultrahigh specific surface area for multiplex functionalization	Non-specific cellular internalization	29,65,88,142,153

CTCs seem to represent an ideal therapeutic target for nanomedicine. Nanomaterials including liposomes and Au NPs have been explored for the direct neutralization of CTCs in the circulation. Many other^119^,^136^ nanomaterials may also prove useful for CTC targeting in the circulation with the purpose of preventing metastasis.
